# Reorganization

**Published:** 1903-05

**Authors:** 


### TEXAS MEDICAL JOURNAL.

AUSTIN, TEXAS.

A MONTHLY JOURNAL OF MEDICINE AND SURGERY.

EDITED AND PUBLISHED BY
F. E. DANIEL, M. D.

ASSOCIATE EDITORS:

WITTEN BOOTH RUSS, M. D., San Antonio, Texas.

P. M. PAYNE, M. D., Brownwood, Texas.

Published Monthly at Austin, Texas. Subscription price $1.00 a year in advance.

Eastern Representative: John Guy Monihan, St. Paul Building, 220 Broadway, New York City.

Official organ of the the West Texas Medical Association, the Houston District Medical Association, the Austin District Medical Society, the Brazos Valley Medical Association, the Galveston County Medical Society, and several others.

**The New Constitution.**

Reorganization. Fall in, Boys! The Texas State Medical Association certainly needs reorganization. The *Red Back* has for years advocated and insisted upon making a change in our constitution and methods, pointing out that, working under the constitution adopted thirty-five years ago, and pursuing the methods then inaugurated, we had utterly failed to organize a State Medical Association embracing even 10 per cent of the physicians of the State. There has been going on a kind of metabolism, constructive and destructive, all these years, a steady taking in and dropping out of members, until today the net gains to the active membership in nineteen years, amounts to just forty-two! Less than three a year! In 1884 (Belton meeting, see transactions 1884) there were on the roll 280 active members. There are today 322. In that time there had been admitted to membership approximately a thousand, and that number, less forty-two, have dropped out and some have died. All readers of the *Red Back* will recall how we have insisted upon trying some other plan, some method of making members, once in, stick. The body has always lacked cohesion. But what the trouble has been, and what the remedy is, I don't know and could not offer a better plan.

But the problem has apparently been solved by the adoption of the new constitution. It seeks to organize the medical profession from the ground up, as it were, beginning with the individual physician, who will be appealed to personally by active members of existing organizations, to join in the procession; to enroll himself in his county society. Membership in the county society entitles him to membership in the State Association and in the American Medical upon the payment in the first of $2 annually, and in the latter $5. But membership in the latter is optional. The county society being the gate-way that leads, without obstruction, all the way up to the National body, should be carefully safeguarded so that no quack or other unworthy individual may be admitted. There are about six thousand "regulars" in Texas. The present membership of the State Association represents 6.4 per cent of that number. Let us all pull together and corral the great majority into one big, harmonious, smooth-working and glorious State Association that means something; an organization whose voice will be heard and heeded by the men sent to Austin to *do the will of the people,* and not their own. We can never hope or expect to enroll them all. There is a large contingent of doctors that could not be induced under any circumstances to join a medical society, not even for a premium. Texas has now fallen in, and aligned herself with the "Great American," and we expect to see that great center attract to itself a majority of the eighty thousand physicans in this "free and enlightened" country. *Then,* perhaps, Congress will listen to the cry that goes up and has ever gone up from them. "Do something to protect the public health!" They may then be made to realize that a Department of Public Health is as important as that of agriculture, at least.

